# Evaluation of the Surface Integrity and Recast Layer in Electrical Discharge Turning of WC-Co Composites

**DOI:** 10.3390/mi15060707

**Published:** 2024-05-27

**Authors:** Mehdi Soleymani, Mohammadjafar Hadad

**Affiliations:** 1 School of Mechanical Engineering, College of Engineering, University of Tehran, Tehran P.O. Box 14155-6619, Iran; soleimani.mehdi@ut.ac.ir; 2 Department of Mechanical Engineering, College of Engineering and Technology, University of Doha for Science and Technology, Doha P.O. Box 24449, Qatar

**Keywords:** electrical discharge turning, electrical discharge machining, EDT, EDM, surface roughness, recast layer, EDX, elemental mapping

## Abstract

Tungsten carbide (WC) and its composites are typically associated with high hardness and high wear resistance, posing challenges in conventional machining processes like turning. To address the machining difficulties of WC-Co, electrical discharge turning (EDT) was proposed. The rotational speed in EDT is a key factor influencing the machining results; however, conflicting reports exist about its impact on the EDT process. Therefore, the effect of rotational speed on three different machining regimes, including roughing, semi-finishing, and finishing, was investigated using energy-dispersive X-ray spectroscopy (EDX), SEM, and roughness tests. Additionally, elemental mapping was applied to illustrate the element distribution on the machined surface. The results indicated that increasing the rotational speed led to a 10% to 17% decrease in the recast layer thickness and a 14% to 54% reduction in the surface roughness (Ra).

## 1. Introduction

Recently, cemented carbide has emerged as a leading choice for tool and wear-resistant part manufacturing due to its exceptional strength and hardness. However, its high hardness and brittleness pose significant challenges for the mechanical machining process, making it an extremely difficult material to work with [1]. Because of WC’s inherent characteristics, conventionally machining it involves high temperatures, a high cutting force, and remarkable tool wear [2]. So, performing ultra-precise machining of tungsten carbides and obtaining superior surface finishes using traditional machining techniques pose significant difficulties. Also, there has been limited progress in achieving accurate and efficient machining of tungsten carbide using ultra-hard tools [2]. Electrical discharge machining (EDM) applies a thermoelectric form of energy to machine conductive materials by melting and vaporizing them. The melting and vaporizing occur by means of numerous discharges between the tool electrode and the workpiece, which is known as gap space. EDM has a wide range of applications, like the automotive industry, dies, aerospace, and surgical components [3]. EDM is able to machine hard materials such as cemented carbide, electrically conductive ceramics, and quenched steel precisely [4]. An experimental investigation was conducted to assess the enhancement of the surface properties of die steels achieved using the powder mixed electric discharge machining (PMEDM) process. The surface finish was examined to observe the variations when machining with Si, W, and graphite powders mixed into the dielectric fluid. Subsequent analysis of the machined surfaces using Scanning Electron Microscopy (SEM) and energy-dispersive spectroscopy (EDS) examined the migration of the elements from the powder, the dielectric medium, and the tool. Factors such as the powder mixed with the dielectric, its concentration, current, and pulse duration were identified as significant in influencing the surface finish. Analysis through SEM and EDS revealed notable material migration from the added powder, electrode, and dielectric to the machined surface [5]. In electrical discharge machining, the material removed from the workpiece is quickly solidified once it is expelled into the dielectric fluid and was typically considered not to reattach to the electrodes. However, researchers have studied how machined material reattaches to the tool electrode surface and elucidated the mechanism behind this reattachment. Following the machining of slots with high aspect ratios, a combination of SEM, EDS methods, single discharge tests, and cross-sectional analysis was employed to demonstrate that debris reattachment to the tool electrode occurs non-randomly and is influenced by its re-melting in the dielectric through the secondary discharge process. Consequently, electrodes may display temporary surface characteristics determined by the properties of the attached material. Furthermore, the deposited material on the tool electrode can provide a protective barrier against the wear induced by subsequent secondary discharges, potentially prolonging the tool’s lifespan [6]. During the process of spark transfer from the tool to the workpiece in EDM, the negative ions from the tool make contact with the work surface, whereas the positive ions from the workpiece make contact with the tool surface. The migration of material between the tool and the workpiece during the EDM process has been investigated. Analysis using energy dispersive X-ray (EDX) spectroscopy revealed the transfer of components of the tool material onto the work surface and vice versa [7].

A special form of EDM which has been developed recently is EDT, which is able to machine difficult-to-machine materials into cylindrical shapes [8]. Some previous studies have concentrated on the recast layer and the surface roughness in the EDT process. The surface hardness, surface roughness, and recast layer thickness of AISI D2 steel were evaluated by integrating magnetic force with EDT. The rotational speed and magnetic flux density had a major effect on the surface integrity. Rotational speed was introduced as a significant factor affecting the EDT outputs [9]. The electrode shape influences the EDT quality. Studying the effect of the workpiece’s rotational speed, the electrode’s shape, and jump down time on circularity deviation, the electrode’s wear rate, and surface roughness showed that the workpiece’s rotational speed and the electrode’s shape were the most influential parameters effecting the surface roughness. The surface roughness decreases when increasing the rotational speed. This is due to better debris flushing due to the workpiece’s rotation and better washing of the debris from the gap area, which can decrease the recast layer and result in a smoother surface. The authors reported that increasing the sparking area between the tool electrode and workpiece increases the capacitive effect between them, which can lead to difficulty removing the eroded particles from the machining gap, so the solidified eroded particles will increase the surface roughness. The circularity deviation of the machined surface was improved by using a curved tool electrode. This is due to increasing the spark volume in the gap area. Moreover, the circularity deviation was decreased by increasing the rotational speed because the material removal per revolution decreased. About the electrode wear, the result showed that increasing the area between the electrode and the workpiece increased the volume of the sparks and thus caused more electrode wearing. Also, the electrode wear increased slightly by increasing the rotational speed due to increasing the energy generated between the electrode and the workpiece [10].

Additionally, there are other studies that have also focused on the MRR in the EDT process. The effects of workpiece rotation on the material removal rate (MRR) and surface roughness were studied, and the results were compared to conventional EDM. The results showed that increasing the workpiece rotation speed leads to better debris flushing in the machining gap, which causes an increase in the MRR. In addition, increasing the rotation speed improved the surface roughness by providing more thorough flushing and decreasing the recast layer formation on the final surface. The surface roughness also improved when rotating the workpiece in comparison to using conventional EDM by decreasing the residual stress on the recast layer due to the more uniform distribution of temperature when rotating the workpiece [11]. The accuracy and precision of EDT was optimized using the Taguchi robust design method. The parameters investigated in this study were the intensity of the EDM machine, voltage, pulse-off time, pulse-on time, servo, and rotational speed. The EDM machine used was an ONA H-300. Additionally, a block of pure copper served as the tool electrode, while cylindrical high-speed steel 1.3255 was employed as the workpiece material. Based on the results, the spindle speed, intensity, servo, and pulse-on time were the parameters that most affected the MRR, and the mean of the MRR increased when increasing the spindle speed [12]. EDT of the titanium alloy Ti-6Al-4V and investigation of the effects of the pulse-on time, peak current, gap voltage, spindle speed, and flushing pressure revealed that the most important machining factors affecting the MRR in this context were the gap voltage, pulse-on time, and peak current. Also, the pulse-on time and peak current affected the surface roughness. This investigation reported that the MRR increased when elevating the spindle speed; however, changing the spindle speed did not have a significant effect on the surface roughness. The authors used a 500 × 300 ZNC Electronica EDM machine, SS-304 stainless steel as the workpiece, and a copper block as the tool workpiece in [13]. The effect of ultrasonic vibration and other input EDT parameters was studied on an AISI D2 workpiece. Among the ultrasonic power, discharge current, spindle speed, pulse-off time, and pulse-on time, the workpiece rotation and pulse current had the most significant effect on the MRR and surface roughness, while residual stress was more influenced by the pulse-on time. The study also reported that the MRR increases with decreasing the rotational speed because when the rotational speed decreases, the discharge heating energy is focused on the workpiece [14]. The effect of the external magnetic field, discharge current, workpiece rotational speed, and pulse-on time was investigated on the MRR and overcut. The magnetic field had the most significant effect on the results. Despite the authors’ belief that increasing the rotational speed would more uniformly distribute thermal energy on the external part of workpiece, causing an increased MRR, they reported that the rotational speed did not have a significant effect on the MRR, but increasing the rotational speed caused an improvement in the dimensional accuracy due to a reduction in point energy [15]. The MRR, surface roughness, and tool wear were studied on AISI L2 steel machined using the EDT process. Increasing the discharge current caused an increase in the MRR, surface roughness, and tool wear. Also, increasing the pulse-on time leaded to an increase in the MRR and surface roughness, while the tool wear decreased. The results showed that increasing the rotation speed caused increased roughness [16]. Also, the reason for the increase in the MRR with an increasing rotation speed was attributed to the better removal of the cut debris, leading to an enhancement in the machining stability [16].

In order to address the technical challenges in conventional machining processes and the high costs associated with its increased hardness and intrinsic brittleness, non-conventional machining processes are increasingly being explored for the machining of WC and its composites (WC-Co), especially for applications where dimensional accuracy and complex geometries are primary requirements. Among the non-conventional methods, electro-discharge machining and electro-chemical machining (ECM) are the only methods capable of machining WC–Co composites [17]. Indeed, hybrid machining methods are often preferred in many processes to leverage the advantages of each machining method [18]. Considering the literature, several considerations confirm the need for more comprehensive study of the EDT of WC composites. The first reason is the scarcity of studies on turning WC composites due to the technical challenges they present. The second reason is that the EDT process could be a promising candidate as a hybrid machining process, yet there are no studies exploring its use for machining WC composites. In addition, there are conflicting results on the effect of rotational speed on surface roughness in the literature. Therefore, we decided to evaluate the results of EDT for WC-Co composites, including their recast layer thickness, their surface roughness, and the element distribution on the machined surface.

## 2. Materials and Methods

**Figure 1:**
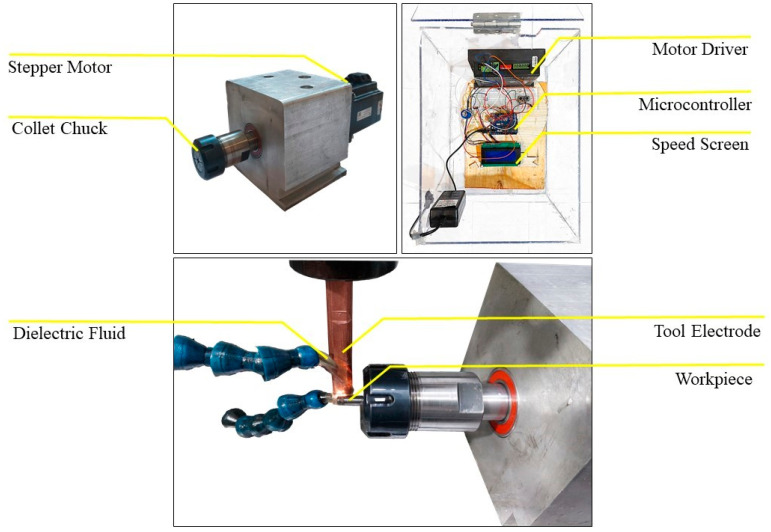
The EDT setup, rotational mechanism, and control board.

To conduct EDT, a rotary mechanism was designed and attached to an EDM machine. This mechanism comprised a stepper motor to provide the rotary power, a coupling to transmit the power to the rotating shaft, a holder to secure the workpiece, the framework, and bearings, as depicted in Figure 1. Also, an Arduino board was utilized in conjunction with the motor driver to precisely control the rotational speed, as shown in Figure 1. Copper was chosen as the tool electrode material, and the workpiece material consisted of WC-Co rods. To perform turning along the workpiece circumference, tool electrodes with a circular cross-section were designed and positioned perpendicular to the workpiece, as illustrated in Figure 1.

**Figure 2:**
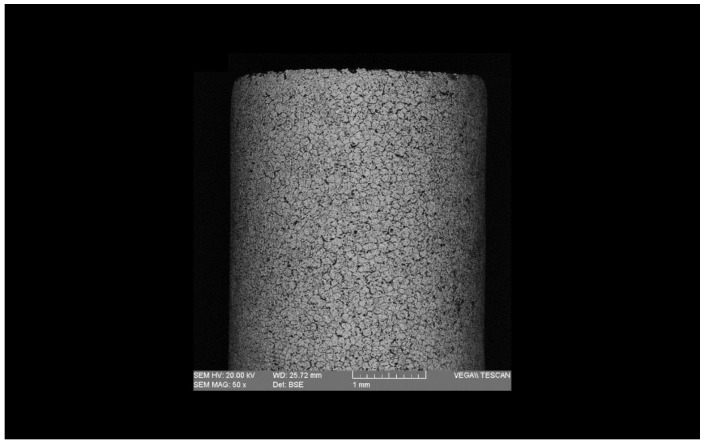
The workpiece machined using EDT.

In order to investigate the recast layer thickness, surface roughness, and element distribution in the EDT process, a series of experiments were designed, as depicted in Table 1. Three distinct machining stages—roughing, semi-finishing, and finishing—were considered for the tests. The rotational speed was varied according to three levels, ranging from 5 to 25 rpm. The feed rate was set to the maximum at 0.5 mm during roughing, reduced to 0.2 mm in semi-finishing, and further decreased to 0.1 mm in finishing. Additionally, the peak current varied from 4 A in roughing to 1 A in finishing. Furthermore, the pulse-on time ranged from 300 µs to 50 µs in roughing and finishing, respectively, while being fixed at 1 µs for all the tests. All the other input parameters remained constant, as detailed in Table 1. Moreover, Figure 2 illustrates the specimen machined using EDT.

## 3. Results and Discussion

**Figure 3:**
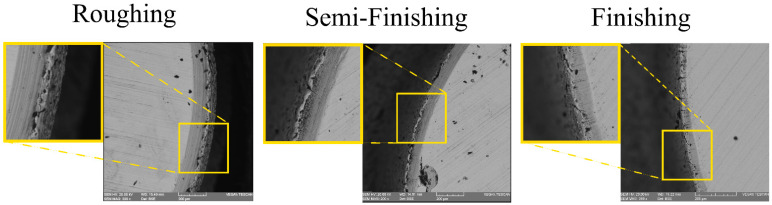
The recast layers in different machining regimes.

**Figure 4:**
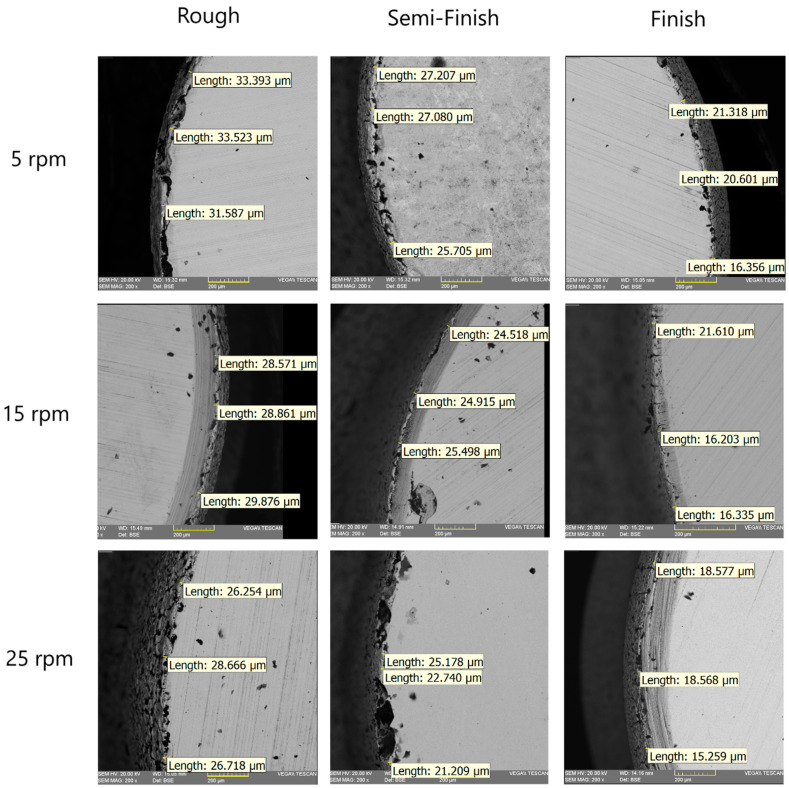
The recast layer measurement at different rotational speeds and with different machining regimes.

**Figure 5:**
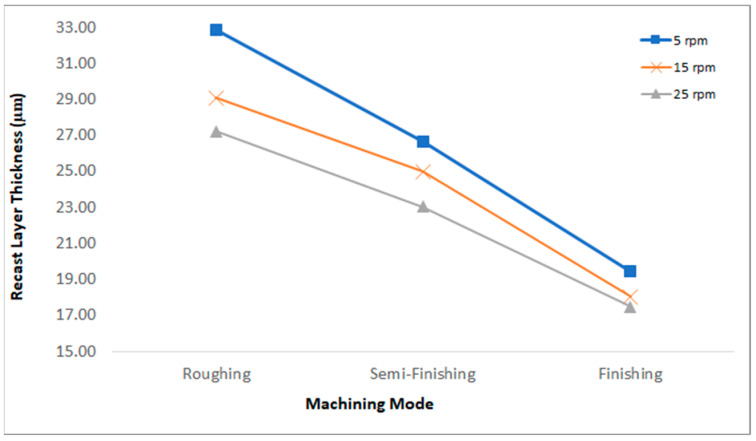
Recast layer comparison at different rotational speeds and with different machining regimes.

To study the recast layer formed during EDT, SEM images were captured of the cross-section of the machined specimens. This method allows for the observation of both the base material (the unmachined part of the workpiece) and the machined area simultaneously. As depicted in Figure 3, the recast layer varies across different machining stages. To compare the recast layer thickness, measurements were taken in three different regions of the cross-section, and the mean value was then compared with the average of the eight other values from the remaining machined specimens. The measured thickness values and their comparisons are presented in Figure 4 and Figure 5.

The molten metal that was not ejected and the redeposited globules solidified on the bulk metal surface, forming the recast layer [19]. In fact, the white layer is defined as the material that is melted by the electrical discharge and then re-solidifies on the workpiece surface. The peak current and the pulse-on time are known as the most significant parameters affecting the white layer’s thickness [20]. Increasing the discharge energy leads to an increase in the recast layer thickness [21,22,23,24,25,26]. One of the input factors that results in increasing the discharge energy and the recast layer thickness is an increase in the pulse-on time [27]. In the machining of WC, increasing the current and pulse-on time causes an increase in the thickness of the damaged surface [28]. In fact, the number of particles produced by electrical discharge that are suspended in the dielectric, the cooling rate of the melted material, and the quality of dielectric flushing affect the recast layer [9]. The rotational speed can improve the flushing of the machining gap and wash away residuals [11]. As mentioned, three different rotational speeds were used in these tests. At each constant rotational speed, the diagram in Figure 5 shows that by changing the machining regime from roughing to semi-finishing and from semi-finishing to finishing, the recast layer thickness decreases. For example, at a rotational speed of 5 rpm, the recast layer thickness decreases from 32.834 µm in roughing to 9.425 µm in finishing. Decreasing the discharge current and pulse-on time leads to a lower discharge energy, resulting in fewer suspended and melted particles that can resolidify on the machined surface and form the recast layer.

Equally, the results showed that in each regime, increasing the rotational speed leads to a decrease in the recast layer thickness. Applying a higher rotational speed in EDT increases the cooling rate of the melted particles, preventing them from resolidifying on the surface. Additionally, increasing the rotational speed improves the flushing and helps wash the particles away from the machining gap. For example, in roughing, increasing the rotational speed from 5 rpm to 25 rpm resulted in a decrease in the recast layer thickness from 32.834 µm to 27.212 µm. Also, in finishing, increasing the rotational speed from 5 rpm to 25 rpm resulted in a decrease in the recast layer thickness from 19.425 µm to 17.467 µm.

**Figure 6:**
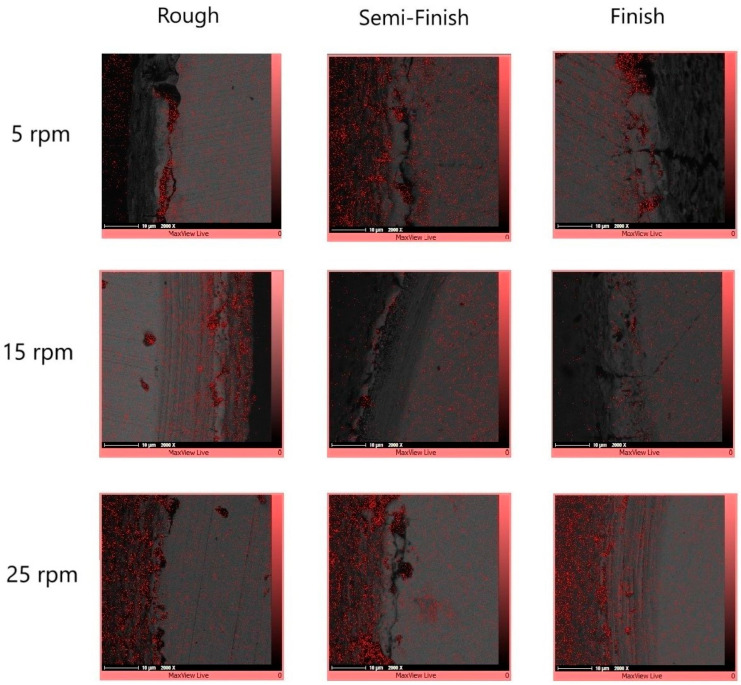
The carbon distribution of the workpiece cross-sections at different rotational speeds and with different machining regimes.

In order to observe the element distribution on the cross-section of the electrical-discharge-turned workpieces, an elemental mapping test was conducted. By superimposing the element map onto the corresponding SEM image, the element distribution of carbon was observed. Figure 6 shows the carbon distribution on the workpiece cross-section at different rotational speeds and for different machining regimes.

**Figure 7:**
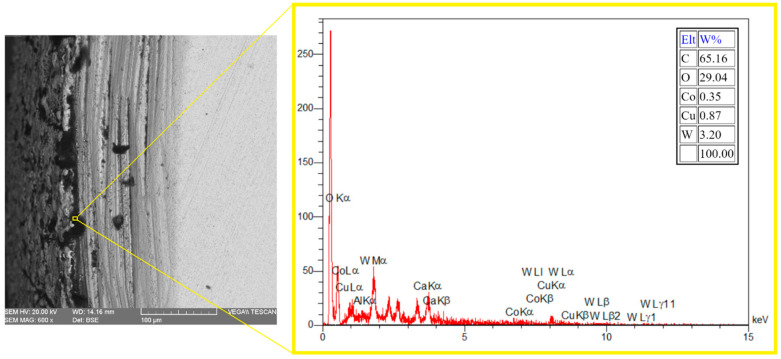
The EDX results in the black area of the machined surface.

During electrical discharge machining (EDM), element migration occurs between the tool electrode and the workpiece, especially when using higher energy discharges. This migration of the materials and their subsequent deposition onto small micro-features can negatively impact product performance. Consequently, this phenomenon is often considered by researchers. The deposition of migrated materials can result in faulty surfaces, as well as surface and subsurface defects [29]. However, carbon migration could be a positive phenomenon when it is deposited onto the tool electrode and decreases the tool wear ratio [30]. It was reported that during the EDM process, hydrocarbon pyrolysis occurs, generating a large population of free carbon particles with negative polarity. These particles are drawn towards the positive electrode under the influence of an electric field, where they are absorbed onto its surface [31]. Figure 6 shows that carbon can be observed in the recast layer, predominantly in the black holes. Due to the presence of numerous and wider holes in the recast layer of the roughly machined specimens, a higher intensity of carbon can be observed there. The EDX test also confirms the high carbon content in the black areas, as depicted in Figure 7.

**Figure 8:**
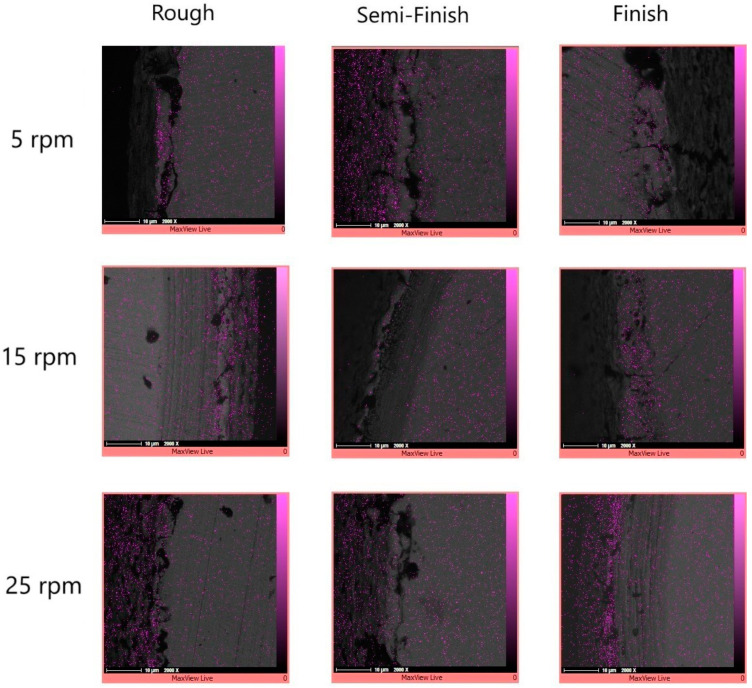
The copper distribution of the workpiece cross-section at different rotational speeds and for different machining regimes.

The elemental mapping results in Figure 8 also showed the presence of copper elements in certain areas, especially in the recast layer and its surrounding region. Copper can migrate from the tool electrode to the workpiece [32], as was the case with copper in our study. The deposition of the melted material can be influenced by either flushing or secondary discharges [6]. When using discharges with a higher energy, the increase in temperature facilitates the easier release of elements, creating free negative and positive ions that can migrate between the tool and workpiece electrodes [7]; however, this is more clear in the later SEM images which have been captured of the machined surface instead of the cross-section.

**Figure 9:**
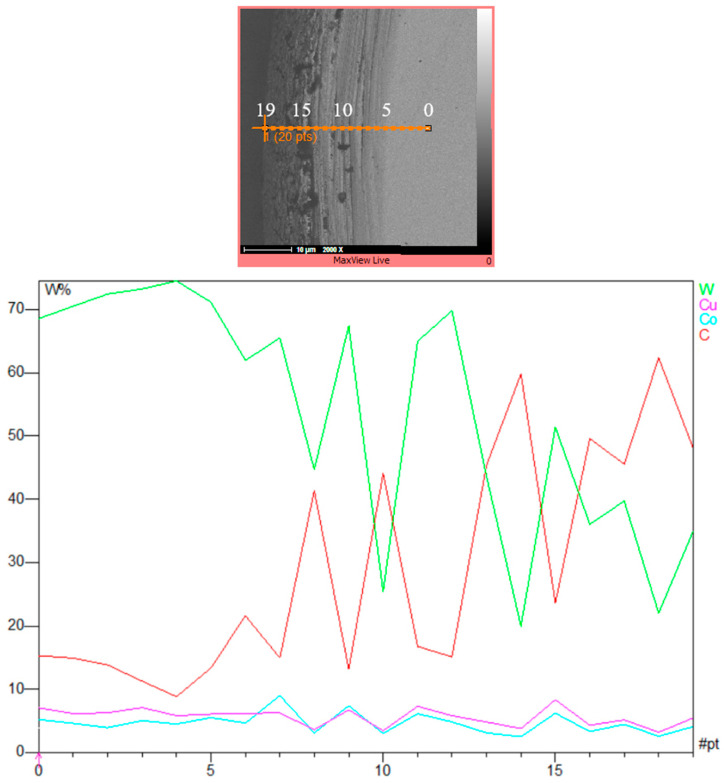
The element distribution along the line.

In order to have a better understanding of the element distribution in the cross-section of the machined specimens, the variations in the elements were evaluated along a line from the original part of the workpiece to the recast layer and the external machined surface, as shown in Figure 9. The results show that tungsten, the primary element of the workpiece, is predominant in the region far from the machining area, although its percentage decreases in the outer areas and the recast layer. Conversely, the carbon element percentage increases from the inner to the outer areas and the recast layer. These findings indicate that during the EDT process, tungsten compositions are replaced by carbon compositions, which may originate from either the tungsten carbide or the carbon in the dielectric [28]. It is noteworthy that there is a slight increase in the copper content in the recast layer compared to the base material.

**Figure 10:**
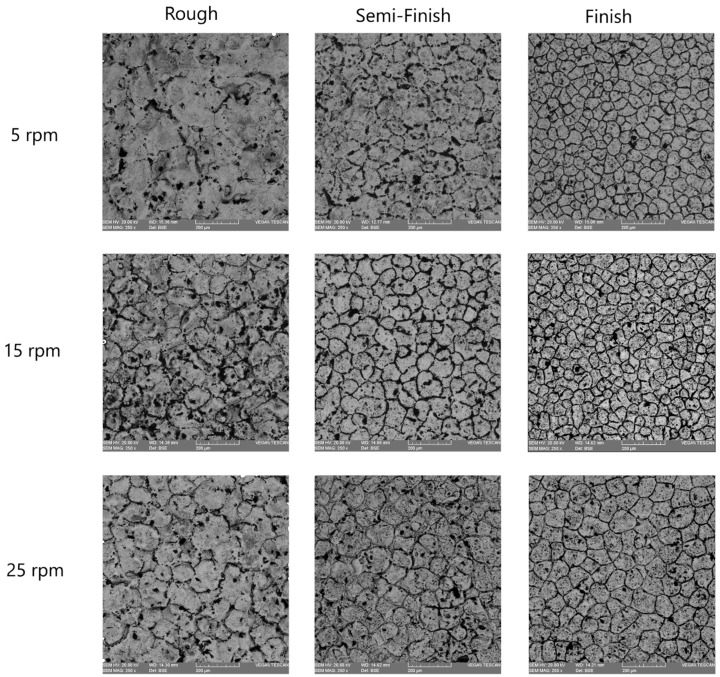
The machined surfaces with different rotational speeds in roughing, semi-finishing, and finishing.

In electrical discharge machining, the surface topography is characterized by shallow craters, spherical particles, melted drops, globules of debris, voids, and cracks, which are created due to the high-heat energy released during the process, followed by rapid cooling [20]. Aiming to evaluate the effect of different rotational speeds on various machining regimes, the surfaces machined using EDT were studied. Figure 10 shows the machined surfaces produced at different rotational speeds using the roughing, semi-finishing, and finishing regimes. A flaky surface is easily noticeable for the rough-machined surfaces where a recast layer exists, while there are no flakes present on the finished machined surfaces. This aligns with previous results showing that the recast layer is less prominent on finished machined surfaces. However, at the lowest rotational speed of 5 rpm, a flaky surface is still visible, which could be attributed to poor flushing of the machining gap.

Additionally, there are more micro-voids on the surfaces machined at higher rotational speeds, a phenomenon that is more prominent for the finishing regime. The melted material withdrawn from the craters after electrical discharge can cover the crater and its surroundings after resolidifying in cases of poor flushing, which typically occurs at lower rotational speeds. Improved flushing also affects the black grid-shaped craters, which mainly consist of carbon elements, as will be discussed in later sections. The black grids fade with an increasing rotational speed and improved flushing conditions.

The SEM images also revealed some micro-cracks on the machined surfaces, which were more noticeable on the rough-machined surfaces. When machining tungsten carbides, electrical discharges with a higher current and pulse time lead to an increase in micro-cracks [28]. As a result, the micro-cracks are more visible on the rough-machined surfaces. Indeed, during the process of resolidifying the melted material and forming the recast layer, the recast layer and the base material cool at different rates due to their varying thermal expansion coefficients. This leads to the formation of tensile stresses in the recast layer, ultimately causing the development of cracks [33]. The occurrence of these cracks was reported when applying electrical discharge to WC-Co composites [28,34].

**Figure 11:**
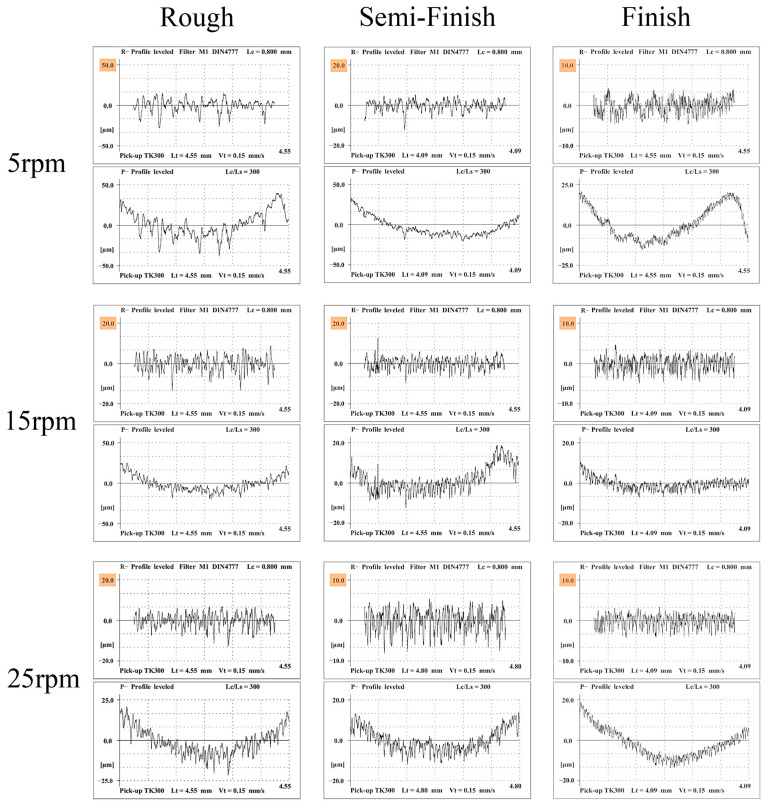
The roughness test results for the machined surfaces with different machining regimes.

In order to gain a deeper understanding of the surface topology of the machined surface, roughness testing was conducted for all the experiments, and the R-profiles and P-profiles have been illustrated in Figure 11.

**Figure 12:**
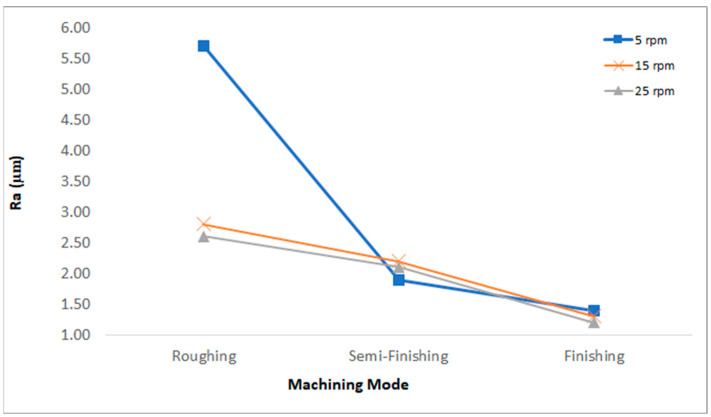
The surface roughness (Ra) results for the machined surfaces with different machining regimes.

The arithmetic average of the absolute values of the profile heights over the evaluation length (Ra), the maximum roughness depth (Rmax) [35], and the average height of the roughness profile (Rz) [36] were considered the two important outputs of the roughness testing. As shown in the Figure 12, the surfaces machined with a lower current and shorter on-pulses have a significantly lower roughness. In EDM, surface roughness is influenced by residual particles, resolidified particles, craters, and micro-voids [32]. Smaller craters and pockmarks formed by low-energy discharges, a low current, and shorter pulse times result in a better surface roughness [28,34,37,38]. Furthermore, in roughing, changing the rotational speed from 5 rpm to 25 rpm significantly improved the surface roughness (Ra), which could be attributed to the better flushing facilitated by the relative speed between the workpiece and the tool electrode [39,40,41], decreasing the number of particles and the resolidified materials. The arc discharges that occur in poor flushing also decrease the surface quality [42]. For the finishing regime, a similar trend can be observed, albeit not as distinctly as seen with roughing. In semi-finishing and finishing, the surface roughness is less affected by the rotational speed. This can be attributed to the decreased impact of flushing during finishing machining due to the reduced machining gap [37,39,40]. Moreover, in almost all the machining regimes, increasing the rotational speed resulted in improved surface roughness. Increasing the rotational speed reduces the focus of thermal energy, leading to a decrease in surface roughness [9].

**Figure 13:**
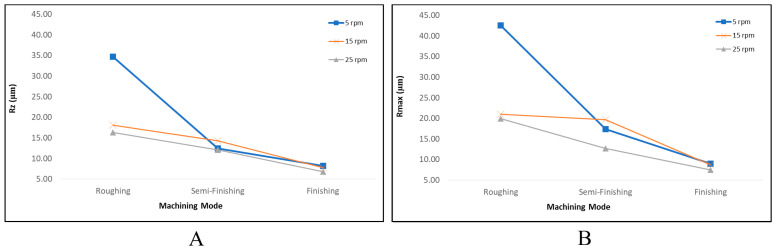
The surface roughness ((**A**) Rz and (**B**) Rmax) results for the machined surfaces with different machining regimes.

Figure 13 displays the Rz and Rmax values for different rotational speeds of EDT across three machining regimes. In rough machining at the lowest rotational speed, i.e., 5 rpm, the Rmax values are significantly higher than the Rz values by approximately an 8 µm difference. Conversely, this difference is around 3 µm for both 15 rpm and 25 rpm within the same machining regime. For the semi-finished machined surfaces, the Rmax values are also higher than the Rz values by 5 µm for 5 rpm, 5 µm for 15 rpm, and 0.6 µm for 25 rpm. However, in the case of the finished machined surfaces, the difference between the Rmax and Rz values is lower, at 0.8 µm, 0.9 µm, and 0.7 µm for 5 rpm, 15 rpm, and 25 rpm, respectively. These results indicate that employing the finishing regime and higher rotational speeds leads to a reduced difference between the Rz and Rmax values of the machined surfaces, suggesting a more uniform distribution of craters and micro-voids on the surface, resulting in a smoother surface.

**Figure 14:**
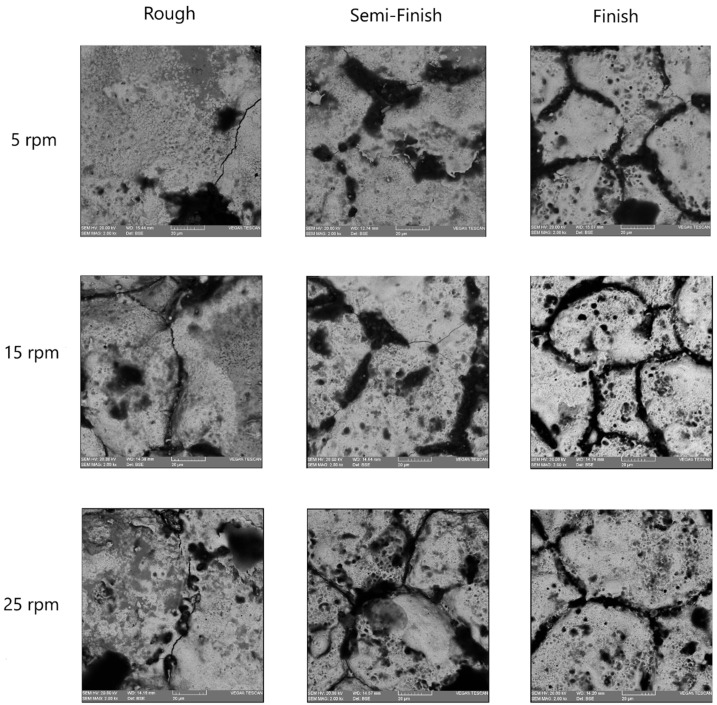
The SEM images of the machined surfaces with different rotational speeds and machining regimes.

To obtain a more precise understanding of the machined surface under different regimes and rotational speeds in EDT, elemental mapping was applied to the machined surface. Figure 14 displays the SEM images with the elemental mapping results superimposed onto them. The surfaces exhibit three distinct areas: black, white, and gray.

**Figure 15:**
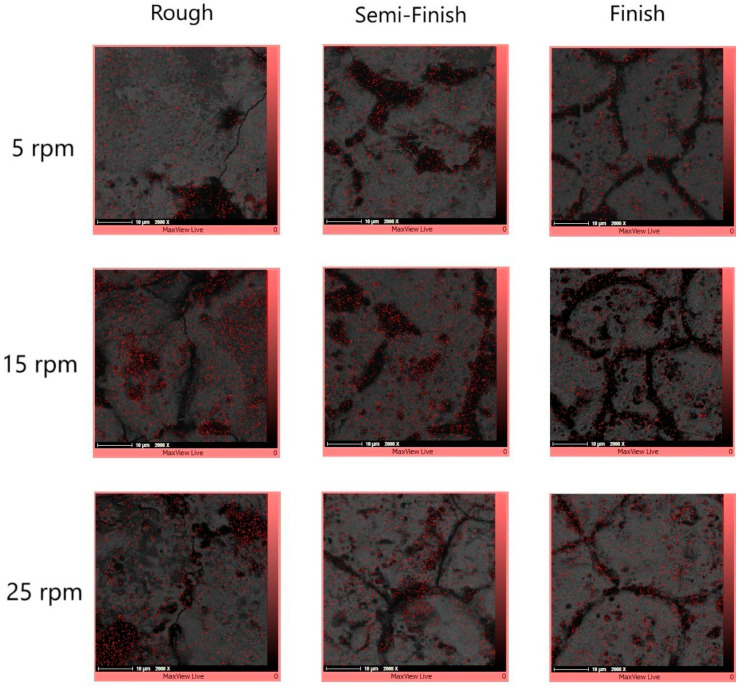
The carbon element distribution on the machined surfaces with different rotational speeds and machining regimes.

**Figure 16:**
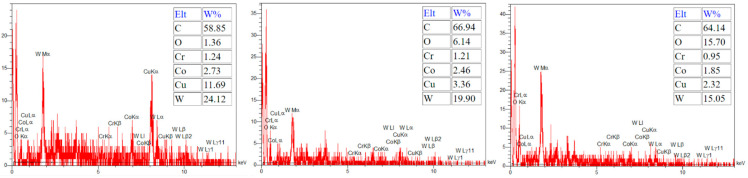
The EDX results on the black areas of the machined surfaces.

Figure 15 displays the carbon distribution on the machined surface. The highest concentration of carbon is in the black area, which predominantly consists of craters and micro-voids. The EDX results in Figure 16 also confirm that carbon is the primary element present in the black areas. During EDM of tungsten carbide, carbon is detected in these black areas, with a portion originating from the dielectric and the rest from the tungsten carbide itself [28]. Indeed, carbon is released during the machining of tungsten carbide by the electrical discharges following dielectric decomposition, melting, and evaporation of the tungsten carbide, especially when there is insufficient flushing. As a result, carbon can be deposited on the machined surface [37]. The presence of relative motion between the tool electrode and workpiece can result in less carbon deposition on the machined surface due to improved flushing, in comparison to die-sinking EDM of tungsten carbide [39].

**Figure 17:**
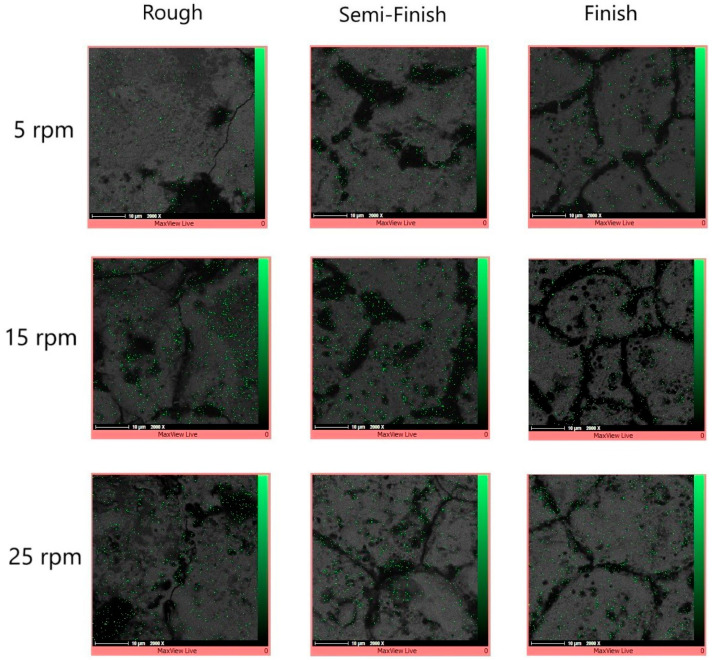
The oxygen distribution on the machined surfaces with different rotational speeds and machining regimes.

**Figure 18:**
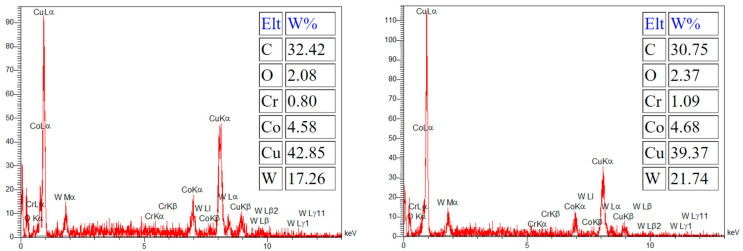
The EDX results on the gray areas of the machined surfaces.

The distribution of oxygen on the machined surfaces is depicted in Figure 17, showing that the highest presence of oxygen is in the black and gray areas. While the existence of carbon in the black areas was confirmed in the previous section, the EDX test revealed that the primary element in the gray areas is copper, as shown in Figure 18. Oxygen can originate from the explosion of bubbles created by the plasma canal, which introduces oxygen and carbon into the machined surface [43]. It was reported that high temperatures during EMD cause oxidation of the workpiece material and migration of this element to the tool electrode. The presence of oxygen is higher in rough machining [7], which can be observed in our results too. It is noticeable that there was a higher deposition of oxygen on the roughing and semi-finishing machined surfaces compared to the finishing machined surfaces. Additionally, the presence of oxygen on the machined surface can be attributed to the oxidation of the melted material during machining, which occurs at high temperatures [27].

**Figure 19:**
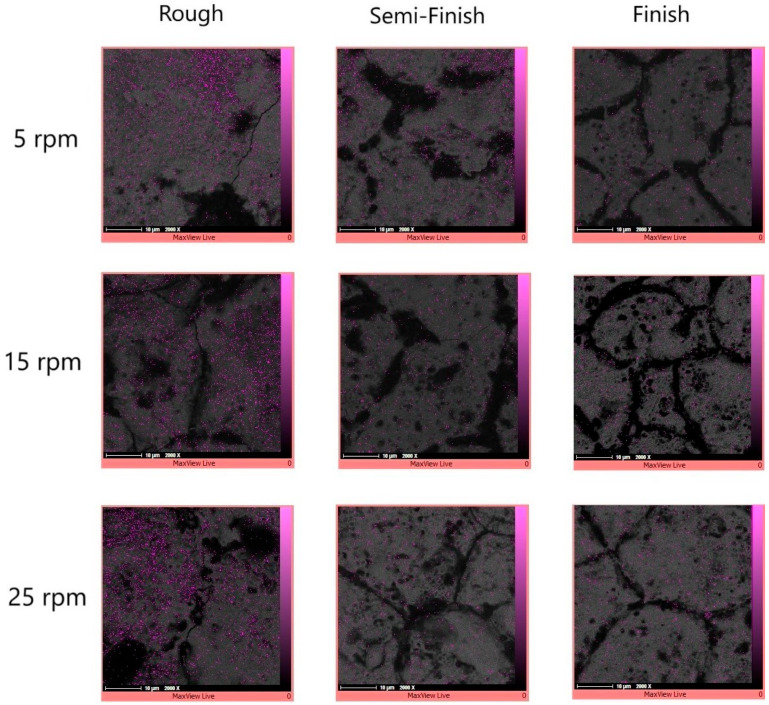
The copper distribution on the machined surfaces with different rotational speeds and machining regimes.

The migration of materials during the EDM process in important because it can lead to resettlement of the materials [7]. The elemental mapping results also confirm that copper is the predominant element in the gray color areas, as shown in Figure 19. The migration of copper occurs from the tool electrode to the machined surface [32], which increases as the pulse time duration and current increase [41]. Applying discharges with a higher energy leads to an increase in temperature and facilitates the easier release of elements, creating free negative and positive ions that can migrate between the tool and workpiece electrodes [7]. According to Table 1, as higher currents and longer pulse durations were used in the roughing regime, the machined surfaces from this regime exhibit more migrated copper elements on them. While some traces of copper can also be observed in the black areas, the concentration of copper in these regions is not as high as that found in the gray areas.

## 4. Conclusions

Aiming to address the technical challenges encountered in the conventional turning of WC composite rods, the EDT process was proposed. Turning of the WC-Co rods was investigated using roughing, semi-finishing, and finishing regimes under various rotational speeds to examine the impact of the rotational speed on EDT machining, while there are conflicting findings in the literature. When turning hard metals such as WC-Co composites for applications in medicine, motors, pumps, and precision measurement industries to produce cylindrical and symmetrical components, it is essential to control machining outcomes such as the recast layer, surface roughness, and elemental distribution. This is crucial to achieving the desired part quality and ensuring machining efficiency. So, following the machining process, the machined parts were evaluated in terms of their recast layer thickness, surface roughness, and element distribution using SEM images, EDX tests, elemental mapping tests, and roughness measurements, resulting in the following findings.

1. The recast layer thickness decreased with an increasing rotational speed due to improved flushing of the machining gap. The reduction in the recast layer thickness was 17% for roughing, 13.8% for semi-finishing, and 10% for finishing when the rotational speed increased from 5 rpm to 25 rpm, indicating a more significant reduction in roughing.
2. Among the two areas in the SEM images, visible as black and gray colors, the black areas mainly comprised carbon, originating from the dielectric fluid and the WC composition. Moreover, oxygen was detected in the black areas, originating from bubble explosions during machining and from oxidation due to high-temperature discharges. Additionally, the gray areas mostly included copper elements migrating from the copper tool electrode, which were more visible using the roughing regime on the machined surface.
3. Based on the surface roughness measurements, the surface roughness (Ra) decreased with an increasing rotational speed due to the lower focus of thermal energy from the electrical discharges and also improved flushing of the machining gap. The decrease in the surface roughness (Ra) was 54% for roughing and 14% for finishing when the rotational speed increased from 5 rpm to 25 rpm. Additionally, comparing the Rz and Rmax values showed that increasing the rotational speed reduced the difference between the Rz and Rmax values, indicating that the machined surfaces had a more uniform topology. When changing the rotational speed from 5 rpm to 25 rpm, the difference between Rmax and Rz ranged from 0.8 to 7.8 µm at 5 rpm, 0.9 to 5.4 µm at 15 rpm, and 0.6 to 3.6 µm at 25 rpm.

## Figures and Tables

**Table 1:** Machining parameters.

No	Machining Regime	Rotational Speed (rpm)	Feed (mm)	Workpiece Diameter (mm)	I (A)	Pulse-On Time (µs)	Pulse-Off Time (µs)	V (V)
1	Roughing	5	0.5	6	4	300	1	80
2	Roughing	5	0.3	6	4	300	1	80
	Semi-Finishing	5	0.2	6	2	150	1	80
3	Roughing	5	0.25	6	4	300	1	80
	Semi-Finishing	5	0.15	6	2	150	1	80
	Finishing	5	0.1	6	1	50	1	80
4	Roughing	15	0.5	6	4	300	1	80
5	Roughing	15	0.3	6	4	300	1	80
	Semi-Finishing	15	0.2	6	2	150	1	80
6	Roughing	15	0.25	6	4	300	1	80
	Semi-Finishing	15	0.15	6	2	150	1	80
	Finishing	15	0.1	6	1	50	1	80
7	Roughing	25	0.5	6	4	300	1	80
8	Roughing	25	0.3	6	4	300	1	80
	Semi-Finishing	25	0.2	6	2	150	1	80
9	Roughing	25	0.25	6	4	300	1	80
	Semi-Finishing	25	0.15	6	2	150	1	80
	Finishing	25	0.1	6	1	50	1	80

## Data Availability

The data are unavailable due to privacy restrictions.
